# Virtual Surgical Planning for Bisphosphonate-Related Osteonecrosis of the Jaw: A Valuable Application in Advanced Cases

**DOI:** 10.7759/cureus.9696

**Published:** 2020-08-12

**Authors:** Harsh Patel, Nariman Saadat, Allen S Ho, Jon Mallen-St. Clair

**Affiliations:** 1 Plastic and Reconstructive Surgery, University of California, Los Angeles School of Medicine, Los Angeles, USA; 2 Oral and Maxillofacial Surgery, Cedars-Sinai Medical Center, Los Angeles, USA; 3 Surgery, Cedars-Sinai Medical Center, Los Angeles, USA; 4 Otolaryngology - Head and Neck Surgery, Cedars-Sinai Medical Center, Los Angeles, USA

**Keywords:** oral reconstruction, bronj, virtual surgical planning, fibula free flap

## Abstract

Bisphosphonate-related osteonecrosis of the jaw (BRONJ) is a rare but potentially devastating complication of extended use of bisphosphonates. fibula free flaps (FFFs) are the gold standard of mandibular reconstruction. Virtual surgical planning (VSP) is a technique that utilizes high-definition three-dimensional reconstructions that enable the production of highly accurate intra-operative surgical guides and templates that help guide osteotomies and fibula contouring.

The aim of this report is to highlight the value of VSP in the surgical management of advanced BRONJ. We report a case study of a woman with advanced BRONJ that required an angle-to-angle mandibular resection with subsequent reconstruction with an FFF. VSP was used to improve the accuracy of the reconstruction and minimize ischemia time.

We present the first reported case of the successful implementation of VSP for the planning of FFF reconstruction for a woman with advanced symptomatic BRONJ that had failed conservative measures.

## Introduction

Bisphosphonate-related osteonecrosis of the jaw (BRONJ) is a rare but potentially devastating complication associated with bisphosphonate (BP) use [1]. BPs are a class of drugs used for the treatment of osteoporosis and malignant bone metastasis through the suppression of osteoclast activity [2]. Risk factors for the development of BRONJ include local and systemic factors. Local risk factors include dentoalveolar trauma or surgery, whereas systemic risk factors include duration of BP therapy, concomitant steroid use, female gender, age, genetic predisposition, obesity, renal disease, low hemoglobin, immunosuppression, and smoking [3-5].

Advanced cases of BRONJ can necessitate segmental mandibular or maxillary resections with free tissue reconstruction. These cases can pose significant surgical challenges due to the extensive, yet poorly defined bone and soft tissue involvement, and often concurrent infectious processes.

Fibula free flaps (FFFs) have become the gold standard of mandibular and maxillary reconstruction [6]. The fibula has a superior quality of bicortical bone that can support dental implants, a long and reliable pedicle, vessel caliber suitable for microvascular anastomosis, and the presence of abundant skin and muscle that can be utilized for soft tissue reconstruction [6]. FFFs have proven to be particularly valuable in the reconstruction of composite mandibular defects, when it is necessary to shape vascularized bone to maintain facial symmetry and preserve oral function [7].

Surgical management of patients with advanced BRONJ is a significant challenge due to the nature of the disease, which often requires a complete resection of all involved areas in order to achieve clinical resolution [8]. Although CT scans allow for greater definition of necrotic foci and neighboring anatomical structures when compared to traditional radiography, it lacks in its ability to provide data on shaping the fibula and gauging the extent of disease [9].

Virtual surgical planning (VSP) provides an effective and advantageous method to traditional CT scans. VSP is a technique that utilizes high-definition three-dimensional (3-D) reconstructions that are translated to stereolithographic models that allow for the production of highly accurate intraoperative surgical guides and templates that help guide osteotomies and fibula contouring [10]. Although relatively new, multiple studies have shown that VSP cases have reduced ischemia time and improved the accuracy of outcomes, with some studies showing reduced post-operative complications and superior patient results [11,12]. Although opponents of the methodology cite the increased upfront cost, others have shown that VSP reduces long-term costs through improved accuracy and shorter operative times [13].

Herein, we present the first reported case of the successful implementation of VSP for the planning of FFF reconstruction for a woman with advanced symptomatic BRONJ that had failed conservative measures. Although other reports describe the use of VSP for various indications of FFFs, we believe that BRONJ is a unique entity that can specifically benefit from the utilization of VSP.

## Case presentation

A 68-year-old female presented to the emergency department (ED) with a past medical history of breast cancer previously treated with surgery and adjuvant chemoradiation, with subsequent prolonged BP therapy yearly for 14 years. She had been directed to the ED by her primary care physician who felt that she required IV antibiotics for her presumed osteomyelitis of the jaw with exposed mandible and recurrent bouts of oral pain and submental swelling that had been refractory to a 21-day course of oral clindamycin. Her prior treatment had also included extraction of her tooth #24 and #25. Mandibular radiographs demonstrated “lytic changes in the anterior mandible”, which prompted a consultation with the Otolaryngology - Head and Neck Surgery service. Historical information was gathered and the patient was diagnosed with BRONJ.

**Figure 1:**
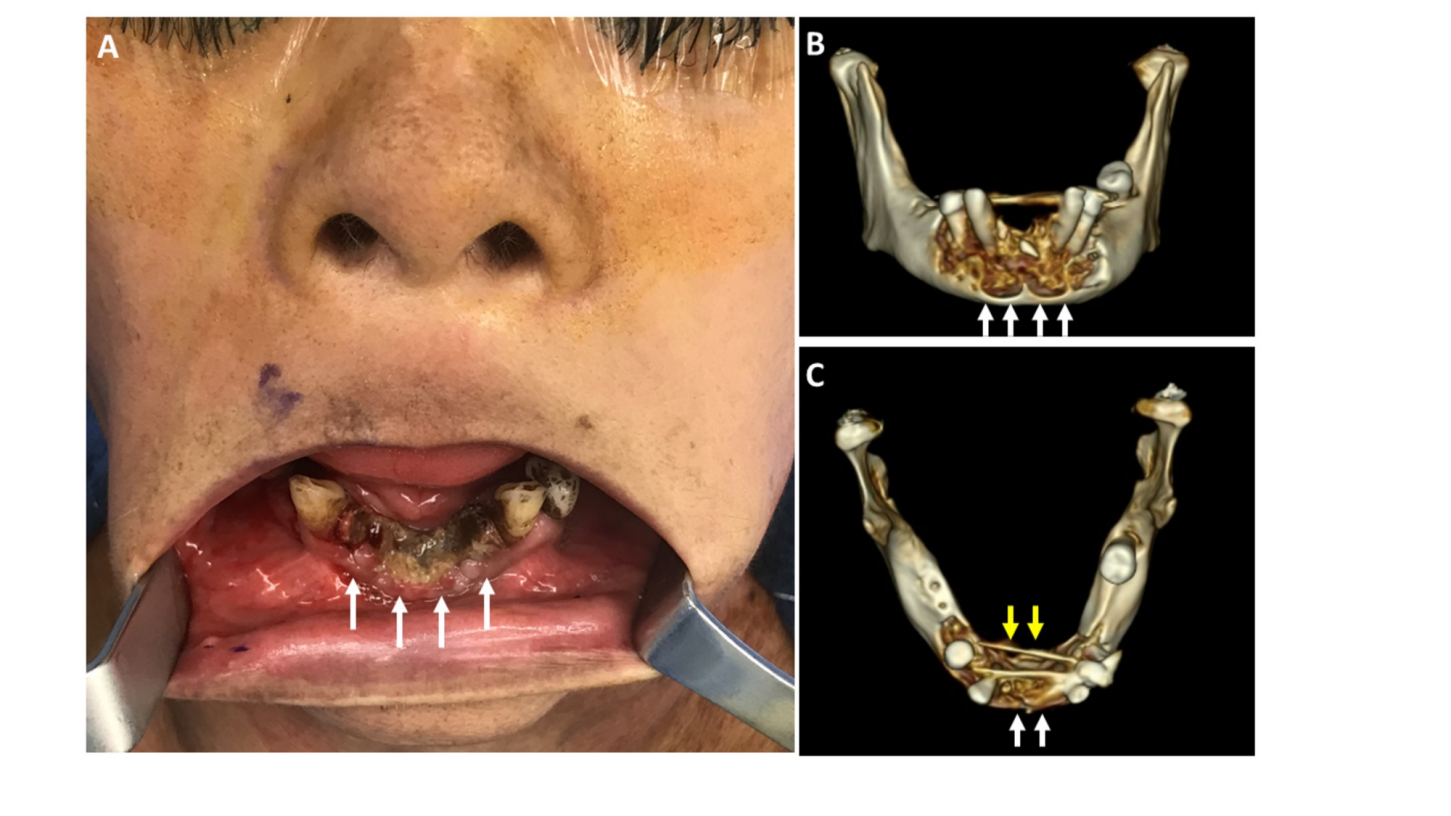
Advanced BRONJ A case of advanced BRONJ, as demonstrated by necrotic exposed mandible highlighted by white arrows (A). A three-dimensional reconstruction of the CT scan demonstrates an extensive erosive process involving the anterior mandible (B), with involvement of the facial (white arrows) and lingual cortex (yellow arrows) (C). BRONJ, bisphosphonate-related osteonecrosis of the jaw

Her physical exam was remarkable for a 4-cm region of exposed necrotic bone, with associated purulence of the anterior mandible corresponding with teeth #22-#27. She had exquisite sensitivity and submandibular pain and swelling. Subsequent CT scan was significant for “necrosis versus sequestration within the anterior mandible, with defects noted on both the lingual and facial cortices” (Figures 1A-1C). IV rocephin and oral metronidazole were initiated but then switched to IV piperacillin-tazobactam for improved antimicrobial coverage. After her symptoms abated, she was switched to oral amoxicillin-sulbactam and then tapered off antibiotics prior to surgery. She was given 24 hours of IV clindamycin following surgery prophylactically. Antibiotics were subsequently stopped as she showed no signs of post-operative infection.

Cultures of the bone were significant for a polymicrobial colonization and pathology of the bone biopsy, and prior debridement was read as “fragments of necrotic bone (osteonecrosis) with clusters of bacterial organisms and foreign material”. With confirmed evidence of BRONJ, and significant pain and chronic infection, it was apparent that untreated her disease would likely progress to a pathological mandibular fracture. The patient was offered a mandibulectomy and FFF given the extent of disease and failed conservative management, which had included dental extraction, debridement, and antibiotic treatment. Her surgery was delayed for multiple months because she required medical optimization due to her past medical conditions for declining liver function. Three months elapsed prior to her receiving medical clearance; CT angiography of her lower extremities thereafter demonstrated patent vessels, normal anatomy of the right lower extremity, and an occluded anterior tibial artery on the left.

**Figure 2:**
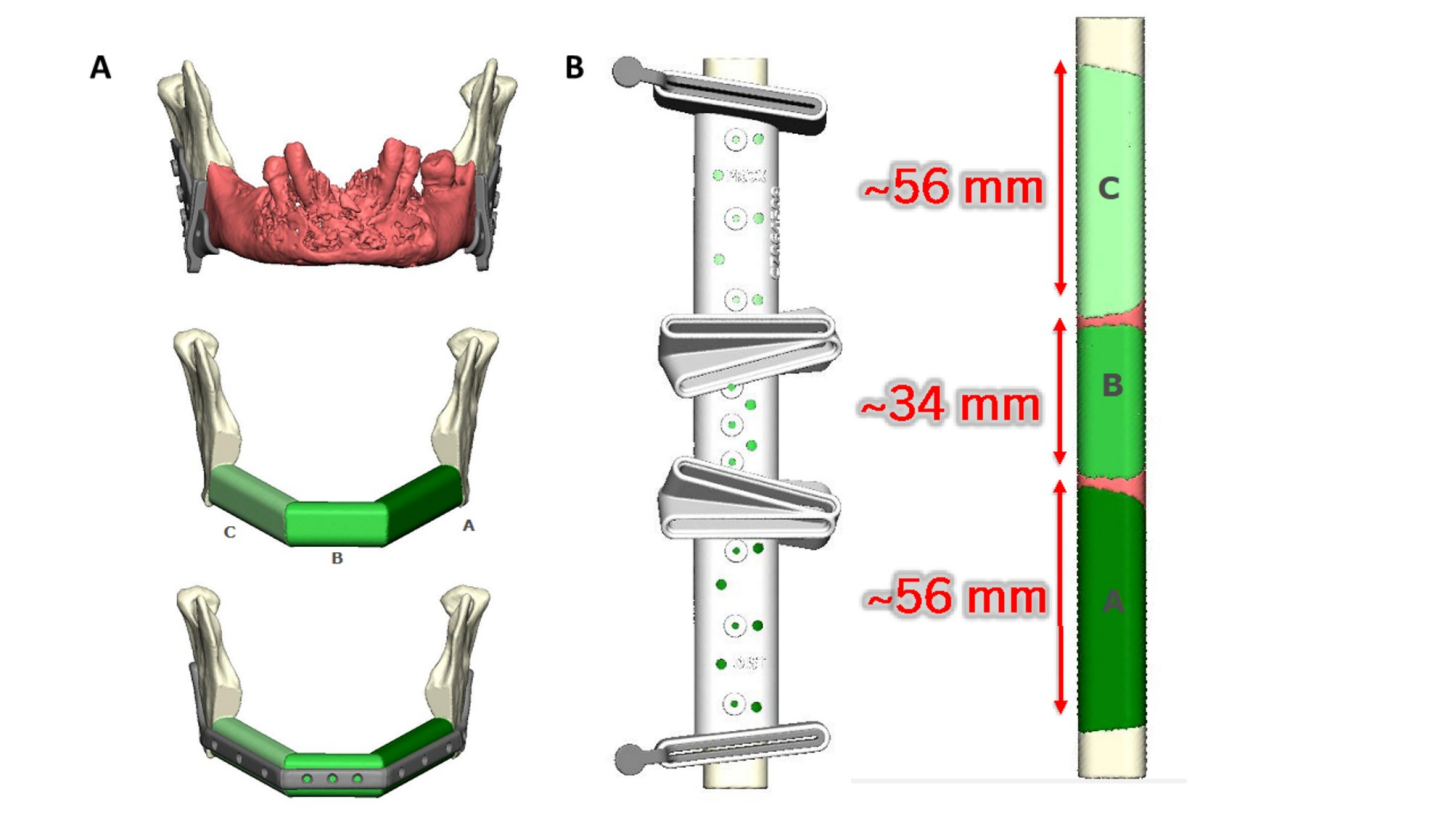
Virtual surgical planning for advanced BRONJ Virtual surgical planning for mandibulectomy osteotomies: plating (A) and closing osteotomies in the right fibula graft to create a three-segment reconstruction of the angle-to-angle defect (B).

Due to the complexity, we felt the case would benefit from VSP. The patient underwent simulation of her mandible and fibula so that cutting guides and osteotomies could be planned for optimal precision (Figures 2A, 2B).

**Figure 3:**
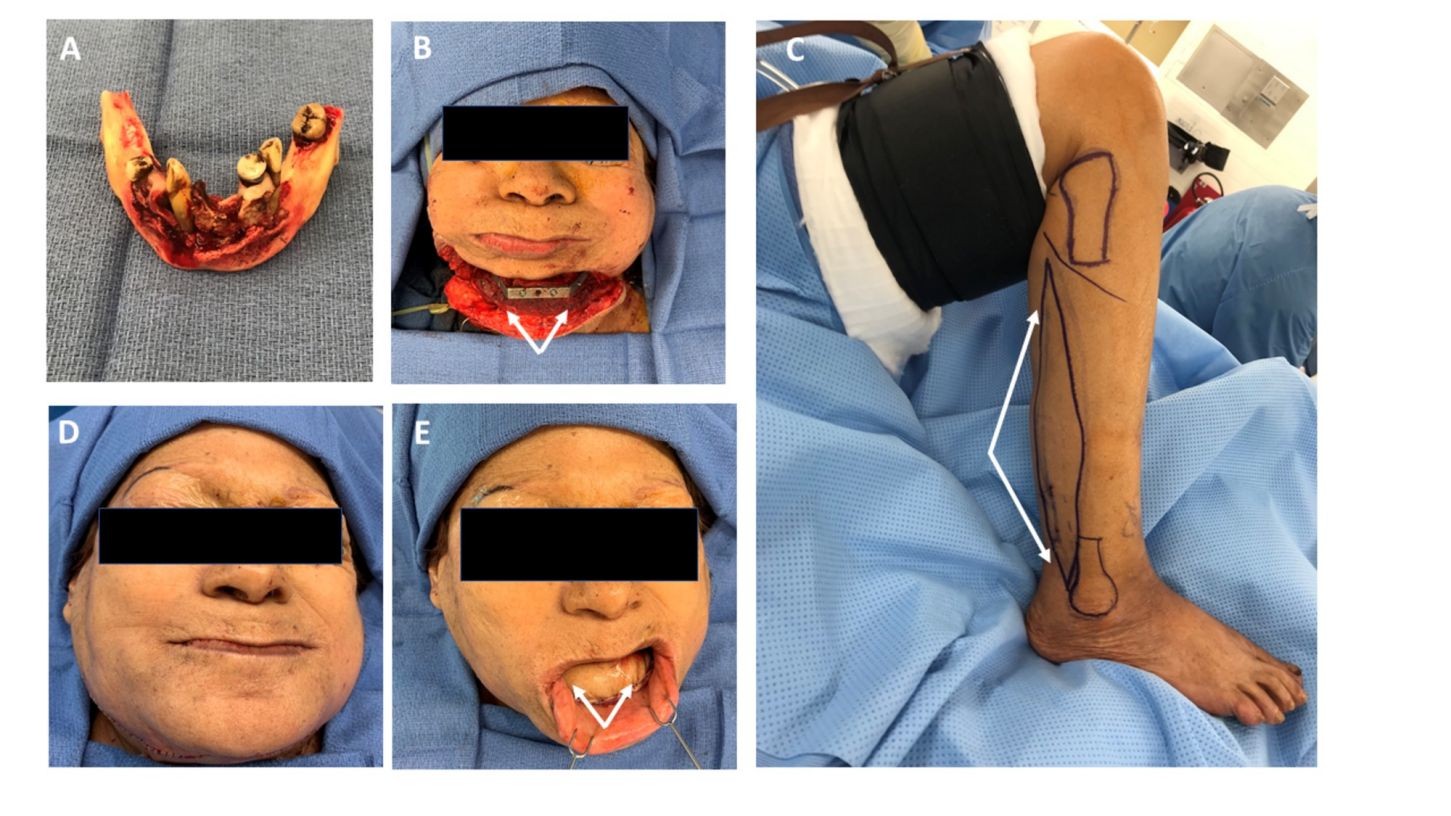
Intraoperative photographs Intraoperative photographs of mandibulectomy specimen with cleared margins (A). The angle-to-angle mandibular defect with graft and reconstruction plate in place (B, arrows demonstrate the reconstruction plate). The FFF skin paddle is planned to restore the mucosal defect (C). Immediate post-operative photos demonstrate adequate projection (D) and appropriate reconstruction of the alveolar ridge mucosa with the fibula skin paddle (E).

She underwent mandibulectomy and immediate reconstruction with an FFF (Figures 3A-3E). She was given antibiotics for 24 hours post-operatively. Her post-operative course was unremarkable; she was decannulated on post-operative day 5, her feeding tube was discontinued on post-operative day 8, and she was discharged on post-operative day 9 after she was able to tolerate a soft diet.

**Figure 4:**
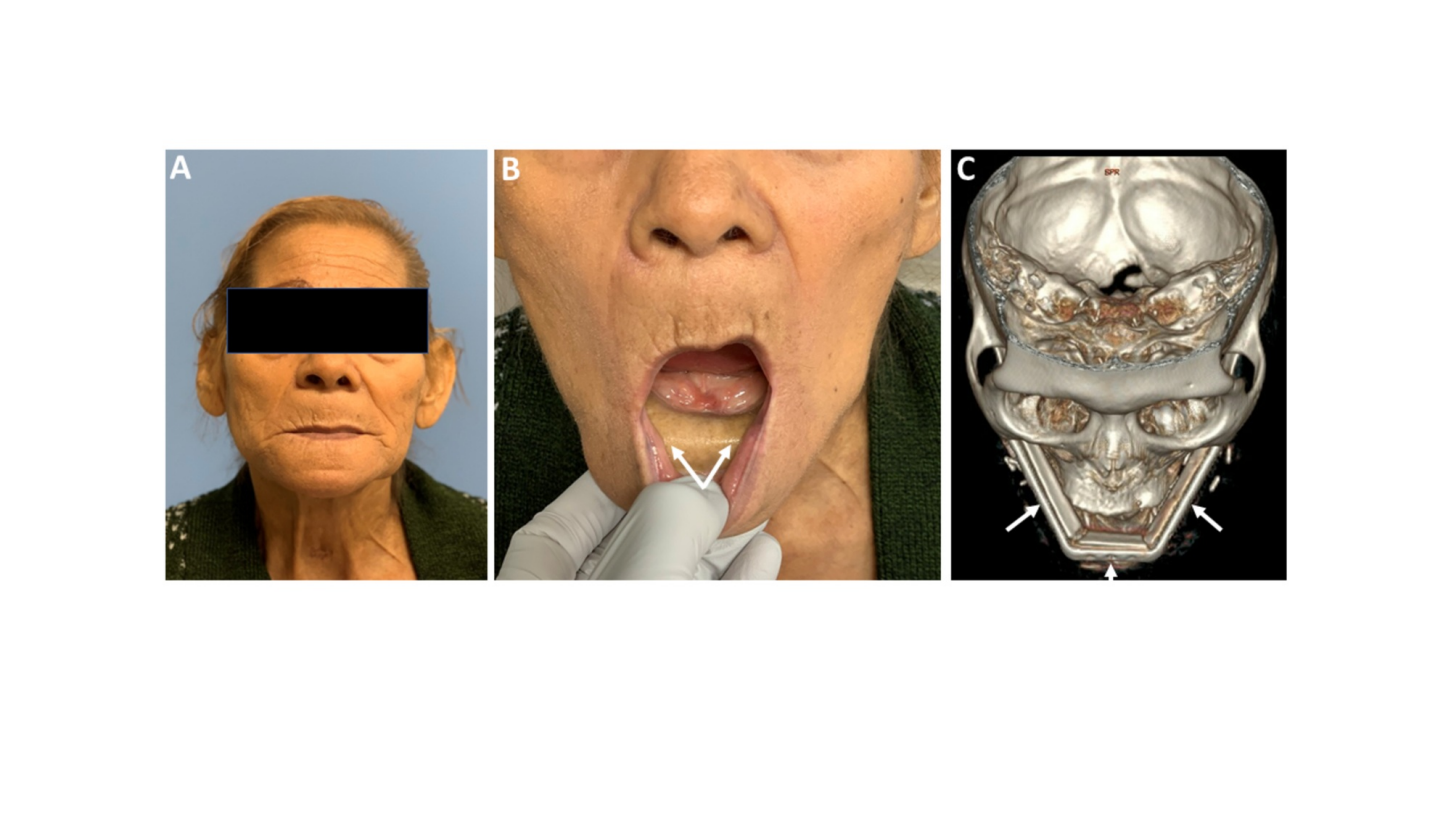
Post-operative photographs following mandibulectomy and fibula free flap using virtual surgical planning Post-operative photographs 12 months after surgery demonstrating adequate projection (A) and appropriate vestibule and alveolar ridge reconstruction with the skin paddle of the fibula free flap (B, arrows demonstrate the skin paddle). A post-operative CT scan demonstrating stability of the graft (C, arrows demonstrate reconstruction plate).

She is currently 16 months out from her surgery, is healing well, and tolerating a soft diet. Her first post-operative CT of the neck demonstrated adequate alignment and stability of the FFF (Figures 4A-4C). She is currently on the waitlist for a kidney transplant due to progressive kidney disease, and dental implants are being deferred until after her transplant.

Surgical procedure

Oral and maxillofacial surgery was consulted for the mandibular resection to allow multiple teams to work simultaneously. An apron incision was planned 2-cm below the inferior border of the mandible. The dissection was carried out through the superficial deep cervical fascia, and the facial artery and veins were identified. The pterygomasseteric sling was incised and the periosteum was isolated anteriorly to expose the inferior border of the mandible from angle to angle. A 10-mm margin around the necrotic bone was identified and incisions were made in the mucosa to isolate the mandible. The 3-D rendered cutting guide was fixed into place by screws, and osteotomies were performed. Intraoperative frozen section returned as “osseous tissue with reactive and regenerative changes and granulation tissue with mixed (predominantly chronic) inflammation with viable bone at the margins”.

For the harvest of the FFF, a 3-cm skin paddle was marked off and dissected through the fascia surrounding the peroneus longus and brevis tendons (Figure 3E). A small muscle cuff was left on the fibula, a guide plate was fastened to the bone with 2-mm locking screws, and the fibula was cut with a bone saw. Cuts were made 7 cm distal to the knee and 7 cm proximal from the ankle. Once recipient neck vessels were isolated and prepared, the flap was harvest from the leg and the microscope was brought in. Prior to anastomosis, a soft tissue defect at the floor of the mouth was corrected, and a free flap skin paddle was fixed with 3-0 Vicryl (Ethicon Inc., Somerville, NJ, USA) using horizontal mattress sutures. An end-to-side venous anastomosis (dominant peroneal vein to the right facial vein) and an end-to-end arterial anastomosis (peroneal artery to the right facial artery) with a 9-0 nylon suture were performed. After confirming patency of the vessels and integrity of the anastomosis, the incisions were closed. Attention was then given to the donor site, which was re-approximated and splinted prior to transfer of the patient to the surgical ward. Total blood loss for the procedure was 300 mL, and ischemia time was 90 minutes. Immediate post-operative images demonstrate adequate projection and appropriate reconstruction of the vestibule.

## Discussion

BRONJ, which is now categorized under the larger entity of medication-related osteonecrosis of the jaw, is a rare complication of BP treatment, with variable incidence in the literature ranging from 0.1% to 10% depending on the medication used [14,15]. Since it was first described in the literature by Marx in 2003, it has been redefined to its current understanding. BRONJ is defined as an area of denuded bone in the maxillofacial region which is present for greater than eight weeks from the time of diagnosis in patients who have been treated with BPs without a history of irradiation to the head and neck region [14]. Of note, the mandible is involved almost twice as often as the maxilla and is thought to be a combined result of an aggregative effect of repetitive microtrauma from mastication in a denser bone in close proximity to a polymicrobial source (i.e., oral cavity) [16]. Since then, the diagnosis of BRONJ has been classified into a three-stage system:

Stage 1: Patient has exposed bone but is asymptomatic and without evidence of active inflammatory or infectious reaction in the adjacent soft tissue.

Stage 2: Patient has exposed bone with associated pain, inflammation, and edema of the adjacent soft tissue with/without a secondary infection.

Stage 3: Patient has exposed bone with associated pain, inflammation, and infection of the adjacent soft tissue that is refractory to oral and IV antibiotics, with an extraoral skin fistula secondary to osteonecrosis of a pathological fracture characteristic of this stage [2].

Recently, a phase 0 has been added to describe high-risk patients exhibiting nonspecific symptomology, with no clinical evidence of necrotic bone.

Historically, osseous microvascular free flaps were not recommended due to concern for nonunion healing deformities [2,14]. Although the pathophysiology is not completely understood, the proposed combination of compact bone due to reduced osteoclast function and availability and the anti-angiogenetic effect of BP is thought to create the ideal environment for secondary bacterial infection in an ischemic territory [17,18]. This theory may underlie why recommendations of osseous free flaps were reserved for the most extreme of cases. A literature review by Neto et al., however, has shown that the reported rate of non-union is relatively low at 5% and has also shown how the procedure has come into favor for patients whose quality of life is thought to be improvable [19]. Pre-operative control of infection with a course of oral or IV antibiotics may be helpful in reducing soft tissue inflammation; however, there is no specific literature regarding peri-operative antibiotics in the surgical treatment of BRONJ.

VSP in head and neck reconstructive surgery is perhaps most applicable in cases of large tumors involving the mandible and adjacent soft tissues, large facial trauma, or osteoradionecrosis (ORN) of the jaw [13]. In each of these cases, the primary defect is complex itself, but the surrounding soft tissue is also heavily involved. In the case of oncologic resections, the need for clear margins highlights the need and limitations of VSP. Through VSP, surgeons are given a 3-D representation of the gross margins of the tumor as well as what boundaries of disease to expect. This additional information is vital in planning of the flap, allowing the surgeon to better estimate the length of bone and soft tissue required to adequate reconstruct the expected defect. This underlines the two-fold benefit of VSP for these cases: aid in the oncologic resection and help to better plan and direct the reconstruction. The drawback, however, stems from the need for clear margins. Although the 3-D reconstruction planning provides greater detail on tumor boundaries in relation to soft tissues than traditional radiographs (i.e., CT), its resolution is limited in comparison to that of the radiograph used to generate the plan. If the resection extends beyond the boundaries of the plan, the cutting guides and generated plans may prove to be inadequate for reconstructing the unforeseen defect. Therein lies the largest limitation of VSP: its inability to be adapted to an evolving, unanticipated surgical field.

ORN poses additional challenges for surgeons that are distinct from an oncologic resection. ORN requires a complete resection, but the extent of the damage may be difficult to appreciate radiographically. Firstly, reconstruction for ORN occurs in the delayed setting and often follows extensive prior oncologic resection and subsequent adjuvant radiation. The two processes not only distort the normal anatomy but also create additional scarring, which make the procedure difficult and obscure boundaries. Furthermore, the majority of head and neck osseous free flap reconstruction rely on the dimensions of a tumor to help shape the flap, without which significant guesswork is required on the part of the surgeon [20]. This leads to numerous trial-and-error attempts intraoperatively, contributing to prolonged operative and ischemia times. VSP helps to limit these obstacles by providing (1) a 3-D model of the surgical bed with precise measurements of the size and shape of the missing elements and (2) allowing the surgery to be conducted in a pseudo-immediate manner with the aid of cutting guides that help alleviate some of the need for anatomical landmarks [10,20].

Though the benefits of using VSP for the management of ORN have been relatively well established, similar advantages are involved in the management of advanced BRONJ lesions that necessitate composite resections with bony reconstruction. Here we report the use of VSP in a case of advanced BRONJ. The authors note that the VSP facilitated the ease of the case and improved the accuracy of the surgical outcome; however, the ultimate value added for using VSP will require further study.

Several case series have demonstrated the utility of microvascular FFF reconstruction in cases of severe or refractory BRONJ, but here we demonstrate the utility of VSP for these cases. Further research will be needed to determine the cost-effectiveness and ultimate value added in utilizing VSP in these complex cases.

## Conclusions

BRONJ is a rarely encountered but significant complication of BP therapy. Our management of BRONJ is evolving, but, in severe cases, the FFF is considered to be feasible and allows a high success rate of re-establishing bony union. We demonstrated the utility of VSP in allowing efficient and accurate reconstruction in these challenging cases.
